# The Long-term Outcome After Resection of Upper Cervical Spinal Cord Tumors: Report of 51 Consecutive Cases

**DOI:** 10.1038/s41598-018-33263-8

**Published:** 2018-10-04

**Authors:** Xin Wang, Jun Gao, Tianyu Wang, Zhimin Li, Yongning Li

**Affiliations:** Department of Neurosurgery, Peking Union Medical College Hospital, Chinese Academy of Medical Sciences and Peking Union Medical College, Shuaifuyuan 1, Dong Cheng District, Beijing, 100730 China

## Abstract

The literature discussing the long-term outcome after resection of upper cervical spinal cord tumors is limited. The purpose of this study was to review the progression-free survival (PFS), overall survival (OS), and long-term outcomes in a consecutive series of 51 patients with upper cervical spinal cord tumors who underwent surgery at our institution between 2005 and 2010. Patient outcome were evaluated using the Japanese Orthopaedic Association score (JOA) and the McCormick functional schema. Follow-up data was collected completely and the median follow-up time was 6.1 years. Gross total resection (GTR) was performed in 27 patients (52.94%) and subtotal resection (STR) in 24 patients (47.06%). Progression-free survival and overall survival at 5 years was 88.23% and 92.16%, respectively. Good prognosis was defined as 74.51% based on JOA scoring. The univariate analysis showed that patients over 60y, tumors located higher than C2, tumor size greater than 4 cm as well as malignant tumors and subtotal resection were factors indicating a poor prognosis. However, the multivariate regression analyses showed only the level of tumor and tumor size were independent risk factors for a poor prognosis. The gold standard treatment for intraspinal tumors is gross total resection and follow-up should be focused on patients with a high risk of poor prognosis.

## Introduction

Primary intraspinal tumors are relatively common, where the overall incidence rate is 0.74–1.5 per 100000 people with 14% of tumors found in the upper cervical region^1–3^. Upper cervical intraspinal tumor refers to primary and secondary tumors located above the level of the C4 segment, which often influences the craniocervical junction (CCJ). Clinical manifestations are variable depending on the location of the tumor, but the main presenting symptom is radicular pain. Tumors in this region can also lead to dyspnea, as well as motor and sensation disorders. Due to the complexity of this anatomical structure, the importance of adjacent structures and the severity of postoperative complications, surgery for the upper cervical spinal canal tumors are considered to be difficult. Moreover, dumbbell tumors are found frequently at this level, making the surgical approach and technique of fixation more complicated.

Due to a dearth of published reports, clinical features and the prognostic factors effecting upper cervical spinal cord tumor treatment outcomes are not clear. The objective of this study was to evaluate long-term clinical outcomes from 51 patients treated for upper cervical tumors via posterior surgical approach. Outcome data was then analyzed for correlations with preoperative variables, treatment methods, resection degree, and histology and there was a median follow-up time of 6.1 years (range 5–8).

## Methods

### Patients

This retrospective study included upper cervical (C1-C4) spinal cord tumors patients who received microsurgery between 2005 and 2010 at the Neurosurgery Department of Peking Union Medical College Hospital (PUMCH). Informed consent was obtained from each patient before surgery. The patient inclusion criteria were: (1) initial surgery for upper cervical spinal cord tumors; (2) long-term follow-up without interruption; (3) no previous cervical spine surgery. Patients with congenital anomalies in the spine such as scoliosis, craniofacial junction malformation and so on, were excluded from the study. A total of 51 cases met the inclusion criteria and were reviewed in this study. The study protocol was approved by the institutional review boards at PUMCH and Peking Union Medical College. All methods were performed in accordance with the relevant guidelines and regulations.

### Demographics, preoperative symptoms and radiological data

Patient datas including age at surgery, sex, presenting symptoms, location of the tumor (surgical level and intramedullary or extramedullary), tumor morphology (dumbbell or non-dumbbell), tumor multiplicity (number of tumors), tumor size (maximal diameter on MR images), surgical resection (gross total resection or subtotal resection), histological diagnosis were reviewed by the same physician. Clinical symptoms were evaluated using the Japanese Orthopaedic Association (JOA) and the McCormick functional schema^4^. The JOA score was divided into 3 main categories, including motor and sensory functions in four extremities and the function of bladder sphincter, with a total score of 17. The lower the score the more severe the deficits. The McCormick functional schema is divided into 4 scoring levels from mild to severe. All patients underwent magnetic resonance imaging (MRI) scan and enhanced examination to determine the tumor location, size and relationship with the surrounding tissue.

### Surgery

The underlying goal of any surgical approach removing upper cervical spinal canal tumors is to remove the tumor as thoroughly as possible without aggravating any preexisting spinal cord injury. For this reason, the posterior approach is generally adopted, such was the case with our patients.

Epidural tumors can be completely removed with a slight separation. In cases where the tumor is wrapped around the nerve root, the tumor is removed as much as possible with the aim of preserving nerve function. Specifically, for treatment of subdural extramedullary tumors, firstly, suspended with thin wire and the dura mater was cut along the midline. Then the tumor was carefully separated from the nerve root and spinal cord. Finally, the blood supply to the tumor was blocked with coagulation, and repeatedly washed with physiological saline; then it was removed completely. If the tumor was large and could not be completely removed, a lump resection or cystectomy was performed to completely excise the tumor.

In order to remove intramedullary tumors, the spinal cord was longitudinally cut from the most prominent and nearest area without blood vessels. The tumor was the separated and removed along its border. When the tumor was separated, we only pulled the tumor not the spinal cord. Dumbbell tumors should be excised from the spinal canal and then excised out of the spinal canal. The outer part of the spinal canal is sometimes closely connected with the vertebral artery, making it difficult to completely cut. Molinari *et al*. maintain that preoperative computed tomography angiography (CTA) or magnetic resonance angiography (MRA) is helpful for finding abnormal vertebral arterie and avoiding vertebral artery injury^5^. Some of our patients had CTA or MRA performed preoperatively to avoid vertebral artery injury.

### Follow-up

Clinical status and radiographs were assessed at follow-up visits through the outpatient clinic. These patients had follow-up periods ranging from 5 to 8 years and JOA scores and the McCormick functional schema were evaluated by the same physician. The recovery rate was calculated as follows: postoperative JOA score − preoperative JOA score)/(full score-preoperative JOA score) × 100. The rate was called excellent (100%), good (60–99%), fair (25–59%), poor (0–24%), or worst (<0%). A rate greater than 60% was identified as having a good prognosis. All MRIs, both preoperative and postoperative, were evaluated by two radiologists.

### Statistical analysis

All statistical analyses were performed using statistical software (SPSS Version 22.0). Summary statistics are provided as percentages and the Wilcoxon rank sum test was used for continuous variables and categorical variables were investigated using the Pearson x^2^ test. A paired student t-test was used for the analysis of preoperative and postoperative JOA score. Moreover, we also used univariate and multivariate regression analyses of variables possibly associated with prognosis. P-values less than 0.05 was considered statistically significant. Finally, PFS and OS analyses were conducted using Kaplan-Meier curves, measuring survival from time of surgery to time of tumor progression or time of death.

## Results

### Preoperative characteristics

In this study 51 patients were surgically treated for upper cervical tumors at the between 2005 and 2010. Table 1 shows the summary of patient characteristics. The median age at diagnosis was 45 years (range 14–70 years), with a female predominance (M/F = 0.82). Two children were also included in this patient cohort. The major presenting symptoms included pain, numbness, muscle weakness, gait and sphincter disturbances, the most common of which was numbness (68.63%). The median follow-up time was 6.1 years (range 5–8).

**Table 1:** Patients characteristics.

Patient’s characteristics	Number	Percent of 51
Number of patients	51	100%
**Sex**		
male	23	45.10%
female	28	54.90%
**Presenting symptoms**		
pain	20	39.22%
numbness	35	68.63%
muscle weakness	27	52.94%
Gait disturbance	9	17.65%
sphincter disturbances	4	8.00%
**Location of the tumor**		
intramedullary	9	17.65%
extramedullary	42	82.35%
**Pathology**		
Neurilemmoma	23	45.10%
Meningioma	6	11.76%
Neurofibroma	6	11.76%
Ganglion cell neurofibroma	6	11.76%
Ependymoma (WHO grade I)	2	3.92%
Astrocytoma (WHO grade II)	2	3.92%
Hemangioma	3	5.88%
Teratoma	2	3.92%
Metastatic carcinoma	1	1.96%
**Tumor multiplicity**		
single	47	92.16%
Two or more	4	7.84%
Tumor size		
More than 4 cm	12	23.53%
Less than 4 cm	39	76.47%
**Tumor morphology**		
dumbbell	28	54.90%
non-dumbbell	23	45.10%
**Surgical resection**		
GTR	27	52.94%
STR	24	47.06%
**Postop improvement rate (JOA)**		
100%	16	31.37%
60–99%	22	43.14%
25–59%	6	11.76%
0–24%	7	13.73%
**Pre McCormick functional schema**		
I	29	56.86%
II	12	23.53%
III	7	13.73%
IV	2	5.88%
**Tumor recurrence**		
yes	6	11.76%
no	45	88.24%

### Tumors and surgical method

**Figure 1:**
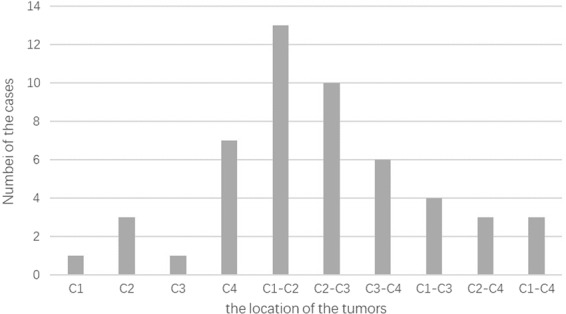
The distribution of tumors according to the location.

There were nine cases of intramedullary tumors and 42 cases of extramedullary tumors including two cases of extradural tumors. In addition, there were 28 cases of the dumbbell tumors, which were classified as type 2A in one patient, 3A in 18 patients, 3B in four patients and type 6 in one patient according to Toyama classification^6^. Tumor site and distribution are summarized in Fig. 1 where most tumors were seen in C1-2.

The statics of histopathology were summarized in Table 1 and the neurilemmoma was the most. All operations were performed by the same surgeon in our hospital. Of the 51 patients, GTR was performed in 27 patients (52.94%) and STR in 24 patients (47.06%). 7 cases of dumbbell tumor excised the facetectomy to expose the tumor. 11 patients were performed with spinal pedicle screw fixation or replantation of lamina or bone graft fusion and internal fixation. Of the 3 patients, one or two nerve roots were removed because of going through an obscure tumor.

The surgical mortality, defined as death within 30 days of surgery, was 0%. The most common complication was chemical meningitis (7/51, 13.73%) recovered by antibodies, which often manifested as fever or headache that responded to steroids. One patient developed impaired wound healing cured by long time physiotherapy. Another patient developed tracheotomy because of pulmonary dysfunction and infection.

### Overall Survival (OS)

**Figure 2:**
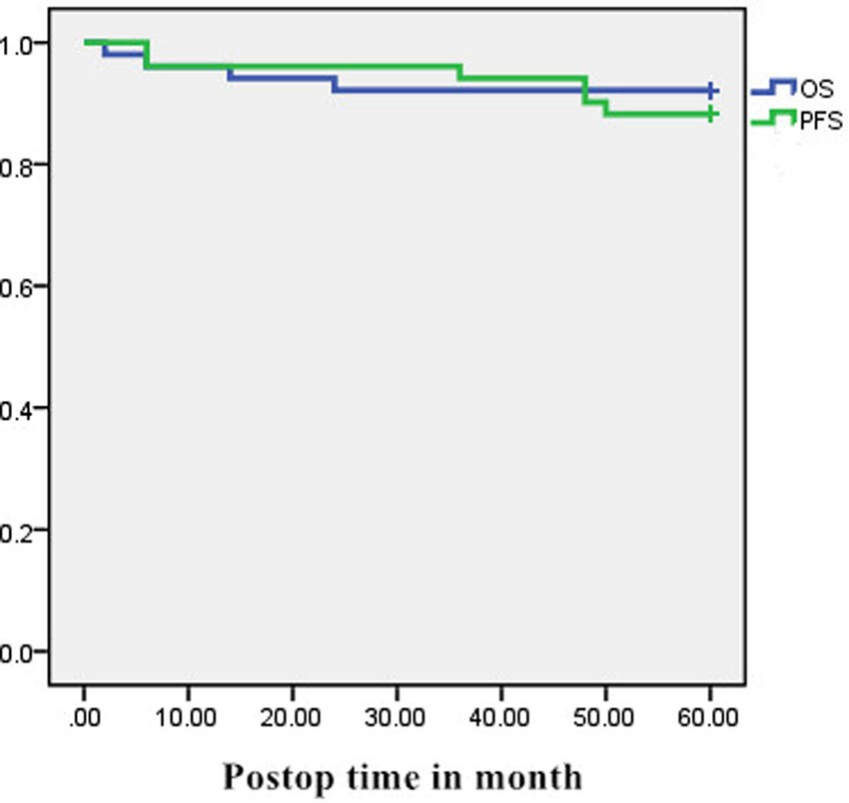
Graph showing Kaplan-Meier estimates of progression-free survival. (green) and overall survival (blue).

The 1- and 5-year OS were found to be 96.01% and 92.16% respectively. Poor preoperative neurological function based on JOA score, malignant tumor and older age at diagnosis might associated with increased risk of death. Four patients included in this study died. The cause of the death of two patients were associated with astrocytoma (WHO grade II), one died due to metastatic carcinoma and the other patient died of advanced age and pulmonary infection (Fig. 2).

### Progression-Free Survival (PFS)

The 1- and 5-year PFS in this material were 96.01% and 88.23% respectively. Six patients (11.76%) experienced tumor recurrence or progression of residual tumor as follows: four local recurrences, one progressive residual tumor and one metastatic recurrence postoperatively. Furthermore, four patients had a second operation to treat recurrent tumors. Tumor recurrence might have been associated with dumbbell tumors, STR or malignant tumors (Fig. 2).

### Follow-up and analysis of prognostic factors

Neurological function was assessed according to the JOA and McCormick functional schema. Table 2 summarizes preoperative and postoperative JOA score, where the mean scores were 12.74 ± 2.07 and 15.28 ± 2.17, respectively. Specifically, there was a significant difference found between the preoperative and fifth year follow-up JOA score (P < 0.001). The mean recovery rate was 67.88% at the time of final follow-up. Table 3 summarized the preoperative and postoperative McCormick functional schema, stating that 12 cases improved, 35 cases were stable, and no cases showed deterioration.

**Table 2:** preoperative and fifth year follow-up score according to the JOA.

	JOA score
**Evaluation time**	
Preoperative	12.74 ± 2.07
The fifth year follow up	15.28 ± 2.17
P value	<0.001

**Table 3:** Neurological function at the preoperative and the fifth year according to the McCormick functional schema.

Evaluation time	I	II	III	IV
Preoperative	29	12	7	2
The fifth year follow up	37	7	3	0

Above all, according to the JOA score, the good prognosis threshold was determined to be 74.51% (38/51). In order to explore the variables possibly associated with prognosis, we adopted both a univariate and multivariate regression analyses (Tables 4 and 5). Univariate analysis showed that people over 60y, tumor located higher than C2, tumor size greater than 4 cm, the malignant tumors and STR were factors indicating a poor prognosis. However, the multivariate regression analyses showed only the level of tumor and tumor size were risk factors for poor prognosis.

**Table 4:** Univariate analyses of variables possibly associated with prognosis.

influence factor		The number of poor prognosis	The number of good prognosis	P value
sex	male	7	16	0.463
	female	6	22	
age	≥60	7	8	0.025
	<60	6	30	
The level of tumor	More than C2	11	10	<0.001
	Lower than C2	2	28	
Tumor size	≥4 cm	8	2	<0.001
	<4 cm	3	36	
Tumor location	Intramedullary	3	6	0.552
	Extramedullary	10	32	
Tumor properties	Benign	9	37	0.016
	malignant	4	1	
surgical resection	GTR	1	25	<0.001
	STR	12	13	
tumor multiplicity	Single	10	36	0.185
	More than one	3	2	
tumor morphology	dumbbell	6	22	0.463
	non-dumbbell	7	16

**Table 5:** Multivariate regression analyses of variables possibly associated with prognosis.

Influence factor	P value	Odds ratio (Confidence interval of 95%)
sex	0.488	0.306 (0.011–8.684)
age	0.348	0.175 (0.005–6.675)
The level of tumor	0.044	0.05 (0.002–1.312)
Tumor size	0.045	0.05 (0.002–1.331)
Tumor location	0.998	—
Tumor properties	0.997	—
surgical resection	0.997	—
tumor multiplicity	0.473	0.186 (0.002–18.409)
tumor morphology	0.998	—

## Discussion

Primary intraspinal tumors accounts for about 14% of tumors in the upper cervical spinal cord^3^. Early clinical manifestations are often untypical nerve root stimulation and compression symptoms, which are easily misdiagnosed as occipital neuralgia, cervical spondylosis, or scapulohumeral periarthritis, among others. However, some patients are not diagnosed until experiencing limb paralysis and bowel dysfunction. Sensory disturbances are the most common symptom during the course of progression, which can be characterized by the numbness of limbs, occipital or facial area, chills, girdle sensation and unstable walking. Furthermore, such tumors can oppress the medulla oblongata structure and cause severe respiratory dysfunction.

The physiological function of the upper cervical spinal cord is considerably important with a complicated anatomical relationship. Compared to other bodily locations, tumors here will cause more severe neurological dysfunction. Most of such tumors are benign and can be permanently cured for life after resection. Once diagnosed, surgery should be carried out as soon as possible to relieve cervical spinal cord compression and promote the recovery of cervical spinal cord function.

In the course of the operation, our team’s experiential suggestions are as follows: (1) every step of separation and resection should be done directly under microscope. In addition, regarding spinal cord hemorrhage, electric cauterization is not recommended. With continuous traction and compression of the spinal cord, it should be protected with cotton pieces during operation. We found it was better to use drip bipolar electrocoagulation to reduce spinal cord injury caused by electric coagulation and heat conduction. (2) Cervical neurilemmoma usually originates from the posterior root sensory nerve and often involves several nerve roots, which should be separated and retained as far as possible, especially when the tumor originates from the anterior root movement. If the tumor is wrapped around the nerve root or unable to be separated from the nerve, it can cut one or two, which is convenient for total resection of the tumor and will not cause obvious neurological dysfunction. (3) Avoid inserting the rongeur forceps into the spinal canal from the middle line. Keep the upper part of the spinal cord when biting the lamina, so as to prevent iatrogenic spinal cord injury. (4) In order to avoid atlantoaxial subluxation or dislocation for tumors removed from the C1 posterior arch and occipital bone, it’s necessary to perform polyaxial screw-rod system and bone graft fusion.

Gottfried *et al*. found that patients who underwent GTR were more likely to remain disease free^7^. In this cohort, only 27 patients underwent GTR. The reason for GTR were as follows: (1) the tumor was too close to the vertebral artery to avoid unnecessary bleeding; (2) vertebral venous plexus hemorrhage caused intraoperative visual field to be unclear; (3) blood supply artery of the tumor was bleeding; (4) intraoperative freezing suggested malignant tumor only underwent decompression operation; (5) for teratoma, some cystic walls were adhered to nerve roots tightly and were unresected; (6) for dumbbell tumors, the tumors in the intervertebral foramen extended far and could not be removed. These reasons for GTR were similar to those discussed by Watanabe M *et al*.^8^.

Complete resection of tumors and restoration of spinal stability are two basic principles for the treatment of intraspinal tumors. Importantly, 36% of pressure on the cervical spine is passed through the vertebral body and intervertebral disc and the remaining strength of the joint is stabilized by a closed ring structure composed of small joints and laminae. Most of the operations required the removal of the spinous process, ligaments, and bilateral laminae and facet joints, especially for dumbbell tumors of the cervical spine. This operation can cause great trauma, resulting in many complications such as posterior process, lateral process and disappearance of the cervical spine.

Two methods have been adopted to maintain the stability of the cervical spine. One is the reconstruction of spinal canal by hemilaminectomy. As the upper cervical spinal canal is relatively wide, it is therefore suitable for hemilaminectomy for tumors located on the side of the spinal cord. Twenty cases of hemilaminectomy were performed in this patient cohort. The other method to maintain stability is the posterior screw-titanium rod fixation technique. In the upper cervical spine, there is no vertebral body at the C1 and the vertebral artery leaves the transverse process at the C2 and the occipital bone is adjacent to the brain and venous sinus. Consequently, the distance between the transverse section of the transverse process and the upper edge of the pedicle should be measured so as to judge the safety of the C2 pedicle screw placement. There were 11 cases of total laminectomy and internal fixation performed among our patient group.

In our study, 11.76% patients had tumor recurrence and 7.84% patients died during the observation period. The PFS and OS at 5 years were 88.23% and 92.16%, respectively. Halvorsen CM *et al*. suggested that neurofibroma, malignant peripheral nerve sheath tumors, subtotal resection, neurofibromatosis, schwannomatosis and advancing age at diagnosis were risk factors for recurrence^9^. In our study, univariate analysis showed that people over 60 y, tumor located higher than C2, tumor size larger than 4 cm, malignant tumors and STR were factor influencing a poor prognosis. However, multivariate regression analyses showed that only the tumor level and tumor size were independent risk factors for a poor prognosis. The difference between the two analyses may be due our limitation to a small sample size in our study. Taken together, our results indicate several factors that should indicate more extensive follow-up for high-risk patients.

Limitations of our study should also be noted. First, it is difficult to guarantee the surgeon charted patient outcomes in a completely unbiased, unambiguous way with regards to JOA score and the McCormick functional schema. Second, the complexity of the upper cervical spinal cord tumors, GTR is very important and Surgical techniques need further improvement. Finally, in this study, we only have 51 patients. Larger cohorts should be performed in our following studies to explore this potential relationship.

## Conclusion

Fifty-one patients with upper cervical spinal cord tumors were reviewed. The PFS and OS at 5 years were 88.23% and 92.16% respectively where good prognosis was considered 74.51% based on post-operative JOA score. The gold standard treatment for intraspinal tumors is GTR but patients of advanced age at diagnosis, tumor located more than C2, tumor size more than 4 cm, or with malignant tumors and STR have a poorer prognosis. Thus, we propose careful follow-up of upper cervical spinal tumor patients with a high risk of poor prognosis.
